# Impaired Structural Connectivity of Socio-Emotional Circuits in Autism Spectrum Disorders: A Diffusion Tensor Imaging Study

**DOI:** 10.1371/journal.pone.0028044

**Published:** 2011-11-23

**Authors:** Stephanie H. Ameis, Jin Fan, Conrad Rockel, Aristotle N. Voineskos, Nancy J. Lobaugh, Latha Soorya, A. Ting Wang, Eric Hollander, Evdokia Anagnostou

**Affiliations:** 1 Department of Psychiatry, The Hospital for Sick Children, University of Toronto, Toronto, Ontario, Canada; 2 Department of Psychology, Queens College, The City University of New York, Flushing, New York, United States of America; 3 Departments of Psychiatry and Neuroscience, Mount Sinai School of Medicine, New York, New York, United States of America; 4 Department of Psychology, The Hospital for Sick Children, University of Toronto, Toronto, Ontario, Canada; 5 The Centre for Addiction and Mental Health, University of Toronto, Toronto, Ontario, Canada; 6 LC Campbell Cognitive Neurology Research Unit and Brain Sciences Research Program, Sunnybrook Research Institute, Sunnybrook Health Sciences Centre, Faculty of Medicine, University of Toronto, Toronto, Ontario, Canada; 7 Department of Psychiatry, Mount Sinai School of Medicine, New York, New York, United States of America; 8 Department of Psychiatry, Montefiore Medical Center, University Hospital for Albert Einstein College of Medicine, Bronx, New York, United States of America; 9 Bloorview Research Institute, Department of Pediatrics, University of Toronto, Toronto, Ontario, Canada; Institute of Psychology, Chinese Academy of Sciences, China

## Abstract

**Background:**

Abnormal white matter development may disrupt integration within neural circuits, causing particular impairments in higher-order behaviours. In autism spectrum disorders (ASDs), white matter alterations may contribute to characteristic deficits in complex socio-emotional and communication domains. Here, we used diffusion tensor imaging (DTI) and tract based spatial statistics (TBSS) to evaluate white matter microstructure in ASD.

**Methods/Principal Findings:**

DTI scans were acquired for 19 children and adolescents with ASD (∼8–18 years; mean 12.4±3.1) and 16 age and IQ matched controls (∼8–18 years; mean 12.3±3.6) on a 3T MRI system. DTI values for fractional anisotropy, mean diffusivity, radial diffusivity and axial diffusivity, were measured. Age by group interactions for global and voxel-wise white matter indices were examined. Voxel-wise analyses comparing ASD with controls in: (i) the full cohort (ii), children only (≤12 yrs.), and (iii) adolescents only (>12 yrs.) were performed, followed by tract-specific comparisons. Significant age-by-group interactions on global DTI indices were found for all three diffusivity measures, but not for fractional anisotropy. Voxel-wise analyses revealed prominent diffusion measure differences in ASD children but not adolescents, when compared to healthy controls. Widespread increases in mean and radial diffusivity in ASD children were prominent in frontal white matter voxels. Follow-up tract-specific analyses highlighted disruption to pathways integrating frontal, temporal, and occipital structures involved in socio-emotional processing.

**Conclusions/Significance:**

Our findings highlight disruption of neural circuitry in ASD, particularly in those white matter tracts that integrate the complex socio-emotional processing that is impaired in this disorder.

## Introduction

Autism spectrum disorders (ASDs) are neurodevelopmental disorders, characterized by impaired social interaction, impaired communication, and repetitive, restrictive, and stereotyped interests, activities and behaviors [1]. Atypical brain growth in young children with ASD prominently affects cortical white matter [2]. White matter is comprised of myelinated bundles of axons capable of rapid transmission of electrical signals between distant brain regions. Efficient conduction of information along cortico-cortical white matter tracts is necessary for the integrated brain activity that, in healthy people, mediates the complex socio-emotional and communication task performance that is otherwise impaired in ASD. To date, several studies have examined the microstructural properties of white matter in ASD, but only a small number have rigorously examined such properties throughout the brain.

Magnetic resonance imaging (MRI) studies have found regional volumetric differences when comparing ASD subjects to healthy controls [3]. Among these brain regions, the frontal lobe is often reported as susceptible by virtue of differences in gray matter volume, white matter volume, or both [4]. Several imaging studies have highlighted white matter and specifically, frontal lobe white matter, as particularly altered in ASD [4], [5], [6], [7]. Interestingly, although broad volumetric differences are prominent and consistent in early ASD, such differences have not been found in adolescents or adults with ASD [4], [5], [8], [9]. More recently, an MRI technique, known as diffusion tensor imaging (DTI) has been used to infer properties of white matter microstructure in the brain in a manner not possible with conventional MRI. DTI based approaches can be used to characterize microstructure in white matter tracts that serve to integrate complex neural circuitry, responsible for the higher-order brain functions that are otherwise impaired in ASD.

Voxel-based, region-of-interest, and tractography-based DTI approaches have been used to characterize white matter abnormalities in ASD brain [10], [11], [12], [13], [14]. Several studies have explored whole brain white matter for evidence of widespread disturbance in ASD using voxel-based morphometry (VBM) style analyses (traditionally used to localize differences in grey matter density). These studies have found evidence of reductions in fractional anisotropy (FA), an index of white matter integrity, across prefrontal [15], [16], [17], superior, middle and inferior temporal white matter regions [11], [15], [18], and the corpus callosum [11], [15] in ASD. However, VBM-style analyses in DTI are limited by misalignment and arbitrarily applied smoothing parameters that can drive abnormal results [19], [20]. Recently, a newer approach, known as tract based spatial statistics (TBSS), that resolves certain methodological issues associated with VBM style analyses and provides an optimized method for voxel-wise comparisons of diffusion properties of white matter [19], has been applied to ASD. This preliminary work has found evidence of widespread abnormalities of white matter in ASD, with some evidence for prominent effects in frontal white matter regions [10], [13], [21], [22]. In addition, TBSS studies indicate that age may have a significant effect on white matter differences in ASD [13], [22], possibly relating to the timing of white matter disruption in the ASD brain. TBSS studies are now providing evidence of widespread white matter differences at the voxel-based level in ASD, however, questions remain regarding whether altered structural connectivity in specific long-range white matter pathways may disrupt neural circuits that relate to the functional impairments that characterize this disorder.

In the present study, we investigated white matter in ASD children and adolescents compared to controls using DTI. Our first aim was to replicate TBSS studies finding widespread white matter differences in ASD using FA, mean diffusivity (MD), axial and radial diffusivity values as DTI indices of underlying white matter microstructure, and to examine whether age, in addition to diagnosis, predicted differences in ASD. Second, we extended previous studies by following up our TBSS findings with tract-specific examinations of select white matter pathways that demonstrated impairment at the voxel-based level. We predicted that frontal white matter pathways that integrate higher-order social and cognitive circuitry would be most altered in ASD, providing further insight into structure-function relationships in this disorder.

## Methods

### Participants

Participants were children and adolescents with full scale IQ>70 as estimated by the Wechsler Intelligence Scale for Children-IV [23]. Children and adolescents with a history of co-morbid psychiatric or medical conditions (e.g., epilepsy), a history of head injury, or of a genetic disorder associated with autism (e.g., fragile X syndrome), were excluded. The ASD group consisted of 19 unmedicated participants, ∼8–18 years of age (3 females; 16 males), recruited through the Seaver Autism Center at Mount Sinai School of Medicine (New York, United States). Diagnoses of Autistic or Asperger's Disorder were made based on DSM-IV-TR criteria and were supported by the Autism Diagnostic Observation Schedule (ADOS) [24] and Autism Diagnostic Interview-Revised (ADI-R) [25]. A diagnosis of Asperger's Disorder was made in 14 participants (2 females, 12 males) without history of language delay. Conversely, Autistic Disorder was diagnosed in 5 participants (1 female child, 2 male children, and 2 male adolescents) with a history of delayed phrase speech (>36 months). Sixteen typically developing control children and adolescents, ∼8–18 years of age (8 females; 8 males), were recruited using advertisements in local media. The study was approved by the Institutional Review Board of the Mount Sinai School of Medicine and informed consent was obtained for all participants as per the Helsinki agreement and institutional guidelines.

### Data Acquisition

All brain scans were performed on a 3-T Siemens Allegra head-dedicated MRI system. DTI scans were acquired using a pulsed-gradient spin-echo sequence with echo-planar imaging acquisition [repetition time = 4100 msec, echo time = 80 msec, field of view = 21 cm, matrix = 128×128, 28 slices, thickness = 3 mm, skip = 1 mm, B-factor = 1250 seconds/mm^2^, 1 non-diffusion-weighted (B = 0) and 12 diffusion-weighted acquisitions, five averages].

### Image analysis

All data were transferred off-line to a Linux-based workstation and processed with the Functional Magnetic Resonance Imaging of the Brain Software library (FSL 4.1; www.fmrib.ox.ac.uk/fsl/) and ANALYZE 9.0 software (www.mayo.edu/bir/Software/Analyze/Analyze) [26], [27], [28]. First, DICOM files of each DTI acquisition were converted into a single multi-volume Neuroimaging Informatics Technology Initiative (NIFTI) file. The diffusion-weighted images were registered to the non-diffusion weighted image by affine transformations to minimize distortions due to eddy currents and simple head motion (eddy_correct) [29], [30], [31]. Non-brain tissue components and background noise were removed using Brain Extraction Tool in FSL 4.1, and quality of image acquisition inspected prior to further processing. The diffusion tensor was then fitted at each voxel [32] using FSL 4.1 Diffusion Toolbox software and diffusion maps produced for FA, MD, and the three eigenvalues of the diffusion tensor: λ1(axial diffusivity map), λ2 and λ3. Radial diffusivity maps were created separately using the image algebra module (λ2+λ3)/2) in ANALYZE.

With respect to DTI indices examined in the present study; FA quantifies directionality of water diffusion on a scale from zero (random diffusion) to one (diffusion in one direction), and MD measures average water molecule diffusion within all three eigenvalues of the diffusion tensor for a given image voxel [33]. Using DTI, diffusion can be further divided into axial and radial diffusivity values. Axial diffusivity provides an index of water molecule movement for the principal eigenvalue (λ1) of the diffusion tensor, and is thought to represent diffusion that is parallel to axonal fibres. In contrast, radial diffusivity is an index of average water molecule diffusion for the second (λ2) and third eigenvalues (λ3) of the diffusion tensor, thought to represent diffusion perpendicular to axonal fibres [33], [34], [35].

As per the TBSS protocol outlined by Smith et al. (2006), and included in the FSL suite, all participants' FA maps were aligned to a pre-defined healthy control target using non-linear registration. Following visual inspection to ensure quality of registration, a mean of all aligned FA maps was used to create a skeletonized image (white matter skeleton), representing the centre of white matter tracts throughout the brain that are common across subjects. As per Smith et al. (2006), the white matter skeleton was thresholded to include white matter voxels with FA values >0.2, in order to suppress areas of low FA and/or high inter-subject variability. Each participant's aligned FA map was then projected onto the mean white matter skeleton, and FA values taken from the nearest relevant tract centre for voxel-wise comparisons.

### Statistical Analysis

#### Whole-brain TBSS Analysis

We first examined all ASD patients and matched controls for the presence of an age-by-group interaction for global DTI indices. For each participant, mean FA, MD, axial and radial diffusivity values for the whole-brain white matter skeleton (produced using TBSS modules in FSL 4.1) were extracted. Univariate analyses of covariance (ANCOVAs) were applied in Statistical Program for the Social Sciences v. 15.0 software (SPSS; Chicago, Illinois), comparing ASD and healthy control groups (diagnosis as between-group factor) for differences on DTI indices (within-group factor) for the whole-brain white matter skeleton, and age-by-group interaction effects examined; partial eta squared estimate of effect sizes were also investigated.

#### Voxel-wise TBSS Analysis

Based on previous structural MRI findings that have shown more pronounced brain differences in children with ASD compared to older individuals [3], [4], [5], [9], and DTI studies indicating that microstructural properties of white matter correlate differently with age in children versus adolescents with ASD [13], [22], [36], three voxel-wise TBSS analyses were performed to characterize between-group white matter differences in: (i) the full study cohort; (ii) children only (≤12 years of age); and (iii) adolescents only (>12 years of age).

Voxel-wise statistics were carried out across participants for each point on the common white matter skeleton using the FSL 4.1 randomize tool. In our full study cohort of children and adolescents, age-by-group analyses were performed to examine whether age, in addition to diagnosis, predicted group differences at the voxel-wise level. Using a step-up hierarchical regression model (designed using the FMRI Expert Analysis Tool, available in the FSL suite) [37] a main effect of group on DTI index (i.e., FA, MD, axial or radial diffusivity) was first measured, with age entered as covariate, followed by testing for regions showing linear interactions between group and age. Non-parametric two sample independent t-tests were used to compare groups of children only and adolescents only. Voxel-wise statistics were based on a permutation approach using the following parameters: t value >3.0; p<0.001 uncorrected; the number of permutations: 5000 without variance smoothing; extent threshold >10 contiguous voxels [19]. These steps were repeated for MD, axial and radial diffusivity values within the white matter skeleton. We only report TBSS clusters from Threshold Free Cluster Enhancement (TFCE) images thresholded at or above 0.95. At this threshold, the TFCE option in FSL only displays white matter clusters that are significant at the p≤0.05 level, fully corrected for multiple comparisons across space using family-wise error [38]. To localize the most prominent white matter differences between groups, clusters that remained significant after thresholding TFCE images at 0.99, to yield multiple comparison corrected results significant at the p≤0.01 level, were also examined. The most probable anatomic localization of each significant cluster was determined using grey matter, white matter and the John Hopkins University white matter tractography atlas [39] tools in FSL 4.1.

#### Post-hoc tract-specific region-of-interest analysis

Although TBSS can provide information regarding white matter at the voxel-based level, this method does not provide information with respect to differences affecting white matter tracts (i.e., tract-based differences), which is important for interpreting the potential impact of white matter disruption on integration within neural circuits. Therefore, the specific long-range white matter tracts that featured the most prominent microstructural differences at the voxel-based level in ASD (i.e., at the p≤0.01 level, fully corrected for multiple comparisons across space using family wise error) were identified, and further tract-specific analyses, performed. First, tract-specific regions of interest (ROIs) were produced based on the John Hopkins University white matter tractography atlas [39], and applied to the mean white matter skeleton, produced using TBSS, and representing white matter tract centres that were common across our DTI sample [19]. Next, skeletonized tract-specific ROIs were used to extract tract-specific DTI indices (FA, MD, axial and radial diffusivity) for each individual subject. Data were analyzed using SPSS v.15.0. Univariate ANCOVAs comparing diagnostic (i.e., ASD, control) and age groups (i.e., children, adolescents) (between-group factors) for differences in white matter tract indices (within-group factor) were performed. Age was added as a covariate in our statistical model and partial eta squared estimate of effect sizes were examined. Where a significant main effect of diagnostic group or diagnosis-by-age-group interaction effect was found, exploratory post-hoc t-tests were performed to identify tract specific differences between groups. Holm's procedure for multiple comparisons was applied as it has higher power compared to Bonferonni correction, while maintaining strong family-wise control of type I error [40]; Cohen's d effect sizes were also reported.

## Results

### Demographics

There were no differences between groups on age or full-scale IQ (Table 1). There was no difference in gender distribution between groups (all p>0.05), using the Fisher's Exact Test, although visual inspection suggested that there were more girls in the control group.

**Table 1:** Demographic Characteristics.

Group		Gender M/F	Age (years)	Statistic		FSIQ	Statistic	
				t	p		t	p
Children & Adolescents	ASD (n = 19)	16/3	12.4 (3.1)	t_1,33_ = 0.03	0.97	98.5 (20.4)	t_1,29_ = −3.4	0.74
	HC (n = 16)	8/8	12.3 (3.6)			100.7 (14.5)		
Children	ASD (n = 11)	9/2	10.2 (1.6)	t_1,19_ = 0.76	0.46	102.1 (19)	t_1,17_ = 0.55	0.59
	HC (n = 10)	5/5	9.7 (1.2)			98 (12.7)		
Adolescents	ASD (n = 8)	7/1	15.3 (1.8)	t_1,12_ = −1.6	0.12	93.4 (22.8)	t_1,10_ = −0.1	0.34
	HC (n = 6)	2/4	16.7 (0.75)			105.6 (17.5)		

Abbreviations: ASD = Autism Spectrum Disorder; HC = healthy controls; FSIQ = full scale IQ.

Data are expressed as mean (standard deviation).

### Statistical Analysis

#### Whole-brain TBSS Analysis

**Figure 1:**
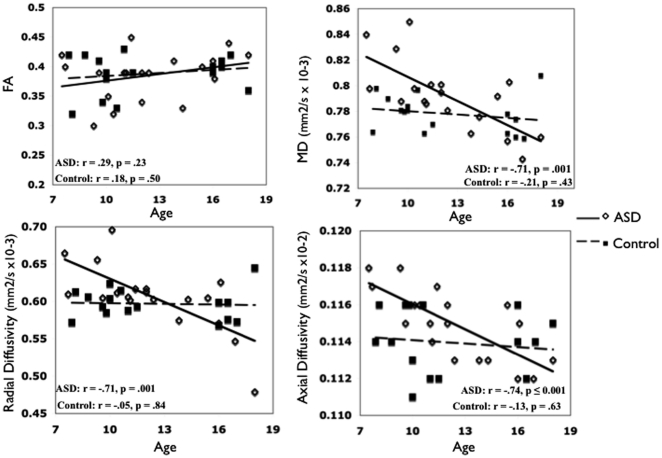
Whole-brain white matter skeleton diffusion properties plotted against age. ASD = Autism Spectrum Disorder; FA = fractional anisotropy; MD = mean diffusivity. Pearson correlations for DTI indices and age are presented.

Univariate ANCOVAs revealed significant age-by-group interaction effects for MD (F_2,32_ = 5.8, P = 0.007, partial eta squared = 0.3), radial diffusivity (F_2,32_ = 4.2, P = 0.02, partial eta squared = 0.2), and axial diffusivity (F_2,32_ = 4.4, P = 0.02, partial eta squared = 0.2), but not for FA (F_2,32_ = 0.96, P = 0.39, partial eta squared = .06), on analysis of the whole-brain white matter skeleton. Linear regression scatterplots for whole-brain FA, MD, axial and radial diffusivity values, each plotted against age indicated significant differences in diffusivity values between ASD and control groups (Figure 1).

#### Voxel-wise TBSS Analysis

TBSS analyses of the full cohort and of the adolescents revealed no group differences in FA, MD, axial or radial diffusivity values. No age-by-group interaction effects were detected at the voxel-wise level in our full study cohort.

**Figure 2:**
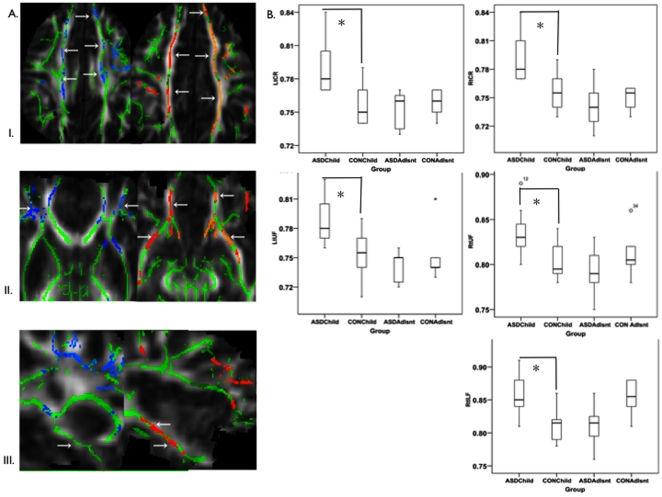
Voxel and tract-based increases in white matter diffusivity in children with autism spectrum disorders (ASD). (2A.) Results of voxel based comparisons depicting white matter clusters featuring increased mean and radial diffusivity in children with ASD. Left: White matter clusters featuring increased mean diffusivity (MD) in ASD are presented in blue. Right: White matter clusters featuring increased radial diffusivity in ASD are presented in red. Rows: (I) left (Lt) and right (Rt) Superior Corona Radiata (CR); (II) Lt and Rt Uncinate Fasciculus (UF); and (III) Rt Inferior Longitudinal Fasciculus (ILF). Note, no difference in MD (image III, left column) was found for Rt ILF in ASD, on TBSS analysis. (2B.) Boxplots depicting MD values for tract-based comparisons corresponding to white matter clusters depicted in 2A. Results for ASD and control (CON) children (Child) and adolescents (Adlsnt) are presented. Significant increases for MD in ASD children were found in Lt and Rt CR (t_1,19_ = 3.1, 3.6, p = .006, 002, respectively), Lt and Rt UF (t_1,19_ = 3.1, 3.1, p = .006,.006, respectively), and Rt ILF (t_1,19_ = 3.3, p = .004). Diffusivity units: mm2/s×10−3. *Significant following multiple comparison correction.

When the analysis was restricted to children (≤12 yrs.), significant widespread increases for MD, and underlying radial diffusivity were seen in ASD across cortico-cortical and inter-hemispheric connections at p≤0.05 (fully corrected for multiple comparisons across space using family wise error). The most prominent white matter differences in ASD (clusters remaining significantly different at p≤0.01) are presented in Figure 2A. At this more stringent threshold, increased MD and radial diffusivity in ASD children were localized primarily to frontal white matter clusters, including: (1) bilateral fronto-medial cortex along frontal regions of uncinate fasciculus (UF) and inferior-fronto-occipital fasciculus (IFOF); (2) frontal pole, within forceps minor of the corpus callosum (CC); (3) bilateral middle and inferior frontal gyrus, within the superior longitudinal fasciculus (SLF); (4) white matter connecting superior frontal and parietal regions, within the superior corona radiata (CR). In addition, increased radial diffusivity only was seen in white matter adjacent to: (1) right temporal cortex, within UF; (2) right temporo-occipital cortex, of IFOF; (3) posterior cingulate gyrus, within forceps major of the CC; and (4) right supra-marginal gyrus through to temporal pole, corresponding to the inferior longitudinal fasciculus (ILF). Therefore, eleven long-range white matter tract domains were identified as featuring prominent microstructural differences at the voxel-based level in children with ASD on our TBSS analysis: bilateral CR, UF, IFOF, SLF, right ILF, forceps major and forceps minor of the CC.

#### Post-hoc tract-specific analyses

**Figure 3:**
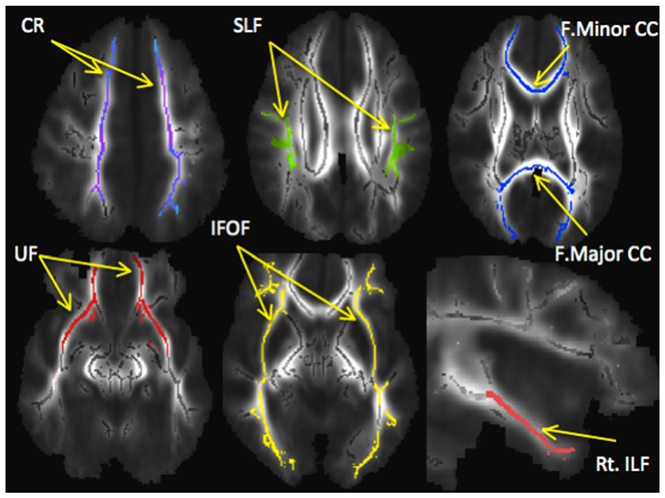
Skeletonized tract-specific regions of interest. Abbreviations: CR: superior corona radiata; SLF: superior longitudinal fasciculus; F.Minor CC: forceps minor corpus collosum; F.Major CC: forceps major corpus collosum; UF: uncinate fasciculus; IFOF: inferior fronto-occipital fasciculus; Rt. ILF: right inferior longitudinal fasciculus.

Tract-specific ROIs were produced based on the John Hopkins University white matter tractography atlas [39] for each white matter tract featuring differences at the voxel-based level in children with ASD (i.e., left CR, right CR, left UF, right UF, left IFOF, right IFOF, left SLF, right SLF, right ILF, forceps major CC, forceps minor CC), and applied to the mean white matter skeleton to create skeletonized tract-specific ROIs (see Figure 3). These skeletonized tract-specific ROIs were then applied to diffusion maps for each participant, and tract-specific DTI indices examined. Significant diagnosis-by-age group interaction effects were found for MD in a number of white matter tracts: right ILF (F_1,30_ = 18.4, p<0.001, partial eta squared = 0.4), left and right CR (F_1,30_ = 12.4, p = 0.001, partial eta squared = 0.3; F_1,30_ = 11.3, p = 0.002, partial eta squared = 0.3, respectively), and right and left UF (F_1,30_ = 9.7, p = 0.004, partial eta squared = 0.2; F_1,30_ = 9.3, p = 0.005, partial eta squared = 0.2, respectively). For radial diffusivity, significant diagnosis-by-age-group interaction effects were found for right ILF (F_1,30_ = 27.9, p<0.001, Partial Eta Squared = 0.5), right UF (F_1,30_ = 12.4, p = 0.001, partial eta squared = 0.3), and right and left CR (F_1,30_ = 10.0, p = 0.004, partial eta squared = 0.3; F_1,30_ = 9.2, p = 0.005, partial eta squared = 0.2, respectively) (Figure 2B). Follow-up t-tests revealed significantly increased MD and radial diffusivity values for tracts featuring significant age-by-group interaction effects in ASD children versus control children only, with substantial effect sizes (e.g. using Cohen's d, for right ILF MD, d = 1.3, and for right ILF radial diffusivity, d = 1.7, for right UF MD, d = 1.2, and for right UF radial diffusivity, d = 1.3). In adolescents with ASD, no significant tract-specific differences were found and effect sizes were considerably smaller (see Table S1 and Table S2).

#### Repeat Analysis in Males only

Although our Fisher's Exact test was not significant when comparing groups for gender distribution, we nevertheless had more females than males in our control group compared to our ASD group. Therefore, we repeated TBSS analyses for MD and radial diffusivity in male children only, in an exploratory fashion, in order to examine whether microstructural differences remained present. Repeat TBSS analysis demonstrated that increased radial diffusivity remained significant at the p≤0.05 level (fully corrected for multiple comparisons across space using family wise error) within left and right UF, left and right CR, and forceps minor of the CC in male ASD children, compared with male controls; increased MD within these regions approached significance following corrections for multiple comparisons across space using family wise error (i.e., p = 0.08). On tract specific comparisons of these tracts, diagnosis-by-age group interactions remained significant for the right UF (MD: F_1,24_ = 5.0, p = 0.04, partial eta squared = 0.2; radial diffusivity: F_1,24_ = 4.7, p = 0.04, partial eta squared = 0.2).

## Discussion

Here, we examined children and adolescents with ASD compared to healthy controls for white matter differences throughout the brain, using TBSS, a voxel-wise comparison method optimized for DTI that is particularly well-suited for microstructural examination of large white matter tracts [19]. We found significant age-by-group interaction effects for whole-brain DTI indices in our sample. Widespread increases in white matter diffusion were found in ASD children but not in ASD adolescents. On voxel-wise comparisons, these increases in diffusivity were restricted to MD (an index of white matter maturation) [34] and radial diffusivity (a more specific index of white matter myelin) [41], [42], [43] across white matter voxels corresponding to cortico-cortical and inter-hemispheric connections. However, at the voxel-wise level white matter disruption localized prominently to voxels within frontal white matter regions. Tract-specific comparisons implicated white matter compromise in pathways that serve to integrate cognitive and social structures in ASD, including tracts that mediate connectivity to frontal and temporal lobes. In particular, alterations found in right UF and right ILF in ASD may signal disruption to a fronto-temporal-occipital circuit that plays a significant role in social and emotional processing.

Our tract-specific findings extend current knowledge of widespread white matter disruption in ASD. Specifically, impairments found in UF and ILF may provide insight into abnormal circuit functions that could relate to production of core ASD symptoms. We also found deficits in the CR, which contains descending fibers from fronto-parietal cortex to subcortical nuclei, and ascending fibers from thalamus to cerebral cortex [44]. Imaging studies indicate that CR impairment may be associated with overall intelligence [45], fine motor control [46], decoding performance [47], numerical operations and mathematical reasoning [48]; domains that have been shown to be altered in ASD [49], [50], [51]. With respect to UF and ILF pathway impairments, disruption of these specific tracts point to interference of structural connectivity within an extended circuit running between frontal, temporal and occipital regions, involved in socio-emotional processing. In particular, we found robust differences in the UF in ASD, a medial white matter tract linking superior, middle and inferior temporal lobe structures (including amygdala and hippocampus) with insular and orbitofrontal cortex [52]. The UF mediates ventral limbic connectivity, facilitating integration between structures that process emotional and cognitive information [52]. Specifically, this tract is thought to play an important role in processing novel stimuli, decoding emotional aspects of auditory information, visual learning, and self-regulation [53]. Concurrent with our findings, a number of diffusion tensor tractography studies have now identified UF abnormalities in ASD [10], [12], [54], [55], [56], [57]. Further, functional imaging studies have implicated disrupted activation and connectivity within regions linked by the UF, both at rest [58], and during socio-emotional performance [59], [60]. Taken together, these findings provide strong support for the presence of UF disruption in ASD and suggest that this disruption may relate to the socio-emotional processing deficits that characterize ASD. Based on our findings, the ILF may also feature impairment in ASD. The ILF extending from occipital cortex, into superior, middle and anterior temporal lobes, mediates connectivity between superior temporal sulcus, which processes biological motion and eye gaze [61], the fusiform face area, responsible for face identification, and the amygdala, involved in processing the social significance of facial expressions [62], [63], [64]. Preliminary diffusion tensor tractography evidence of ILF disruption in ASD [57], [65], and disturbed connectivity among regions linked by the ILF [60], [66], highlight the need for further characterization of this pathway in ASD. Increased MD, and radial diffusivity in UF and ILF may signal impaired transmission along these tracts and secondary disruption to coordinated activity among circuit structures that together mediate decoding of social stimuli, face processing, emotion recognition and regulation; core domains of social impairment in ASD.

On voxel-wise TBSS analysis, our white matter findings localized prominently to voxels within frontal white matter regions in children with ASD. These findings align with accumulating evidence from structural MRI studies implicating pronounced abnormalities in frontal white matter volume among children with ASD [4], [5], [6], [7], [67]. Our work also aligns with functional imaging studies highlighting reduced activation of frontal regions across social and cognitive tasks [59], [68] and reduced functional connectivity with frontal regions in ASD [60], [69], [70], [71], [72]. In addition, our findings concur with recent histopathological data showing structural alterations to frontal white matter connections that signal altered white matter development in ASD, including decreased myelin thickness surrounding axons in the frontal lobe [73].

Typical development of white matter is characterized by increases in FA, and decreases in MD, radial diffusivity, and to a lesser extent axial diffusivity [41], [74], a profile that emerges with white matter myelination, reduced brain water, greater organization of fiber tracts, and decreased extra-axonal space, during white matter development [33], [34], [75]. Increased MD points to increased average diffusion of water in white matter regions. In the present study, differences in global white matter indices in ASD included increased MD, radial and axial diffusivity in younger individuals within our sample, a diffusion profile consistent with immature white matter [41], [74]. Further, our voxel-wise findings indicated that increased MD in ASD was driven by greater radial diffusion, an index that has been related to worsening demyelination on post-mortem investigation of cervical spinal cords affected by multiple sclerosis, and may signal prominent impairment in myelin structure [43]. Other lines of ASD research provide further support for myelin disruption in ASD. For example, a study characterized mice with a disruption to Slc25a12, a susceptibility gene for ASD that encodes for the mitochondrial aspartate-glutamate carrier AGC1. The authors found specific reductions in myelin basic protein positive fibers indicating altered myelination in knockout mice, and reduced myelin-associated glyco-protein in male heterozygotes [76]. Recent evidence indicates that typical white matter maturation relates to acquisition of higher-order cognitive and emotional processes, a relationship that may be influenced by the effects of myelination on regulation of the speed and timing of signal transmission along white matter tracts [77], [78]. Tight control over the timing of signal transmission is posited as critical to synchronous activation of distant cortical regions that work together for optimal mental performance [78], [79]. Signs of broad structural impairments in white matter organization, and myelin, found here, may therefore relate to consistent findings implicating decreased synchrony within neural circuits in ASD [69], [71], [80].

Our findings of broad white matter impairments in ASD align well with previous TBSS studies highlighting widespread microstructural white matter differences in ASD, with prominent effects in frontal white matter voxels [13], [21], [22], [81]. Particularly consistent with our findings are the results of a recent TBSS analysis indicating increased MD and radial diffusivity affecting an array of white matter regions, including white matter voxels corresponding to UF, CR, and ILF domains [13]. However, unlike in our study, others have implicated decreased FA, indicating reduced white matter integrity, in ASD [13], [21], [22], [81]. FA is primarily determined by axonal membranes and secondarily by myelin [34]. Postmortem ASD data has shown increases in axonal density (which aligns with our finding of increased axial diffusivity) and decreased myelin thickness, aligning with our findings of increased radial diffusivity [73]. One could postulate that an increased barrier to diffusion due to increased axonal density, and a decreased barrier to diffusion due to decreased myelin thickness may create little difference in FA between groups. The differences in diffusion parameters in our study were found in ASD children compared to healthy controls, but not in adolescents. Others, however, have found diffusion differences in white matter in both children and adolescents with ASD compared to healthy controls using TBSS [13], [22]. The lack of difference found in adolescents is unlikely to be due to low power, as effect sizes for tract-specific white matter differences in children were considerably larger than those in adolescents. That white matter differences were found in children only in the present study is not surprising given the findings of previous imaging studies, including studies of white matter, showing prominent differences in ASD children, and more subtle white matter aberrations in older individuals with ASD [3], [4], [5], [9], [82]. Based on these results, it has been proposed that brain differences in ASD may be most pronounced closest to the timing of brain insult, when symptomatology may first become apparent [3], [5]. This may be especially true in high functioning ASD (IQ>70), where brain differences compared to controls may be less evident than in lower functioning individuals. Therefore, it may be difficult to detect subtle differences in high functioning ASD as the brain continues to develop in adolescence, especially if alterations begin to normalize, as suggested by the slope of the regression line found here for diffusion parameters when averaged across the whole-brain skeleton (Figure 1).

### Limitations

An important limitation of our study is that our participants were mainly higher functioning individuals with ASD (indexed by average intelligence); therefore, the neuroanatomical correlates of ASD within this subset of individuals may not generalize to all individuals on the autism spectrum. Nevertheless, we would anticipate that more severely affected ASD patients might have even more prominent brain disruption. Secondly, we did not study very young children (i.e. <5 years of age) with ASD, where possibly even more pronounced differences might be anticipated using DTI. Third, while we did not detect differences in white matter between the adolescent groups, our sample size was relatively small, and therefore, further research is needed to characterize white matter in this group. Moreover, although our groups did not differ significantly in terms of gender distribution, there were more females in our control group than in the ASD group. Repeat TBSS analysis for males only was carried out to mitigate concerns regarding gender-based effects on diffusion measures within our sample. Further, although several studies indicate a link between radial diffusivity and myelin [41], [42], [43], changes in this DTI measure can occur with registration error, and in areas of crossing fibers [35], therefore, biological interpretations must be made cautiously. Finally, our imaging acquisition protocol included a 1 mm skip, which could interfere with tract-specific comparisons using a streamline tractography approach [83]. Based on this limitation, a region of interest rather than streamline tractography approach was chosen for tract-specific comparisons in the present study.

### Conclusion

In summary, we found widespread white matter disruption in ASD that might be related to impaired white matter organization and abnormalities in myelination. Impairment was most prominent in frontal white matter voxels (at the voxel-wise level) and along specific white matter tracts that integrate a distributed fronto-temporal-occipital circuit that is integral to socio-emotional processing in children with ASD. Altered white matter tracts in ASD may cause impairment in long-range transmission that in turn leads to secondary interference in the coordinated brain activity that is required for complex socio-emotional processing. The growing evidence for disruption of white matter tracts in ASD that is now provided in several investigations, including our own, highlights the importance of white matter deficits as a core neural substrate of disease. However, further research is needed to elucidate the onset and trajectory of abnormal development of white matter tracts in ASD and better define how these deficits relate directly to clinical and behavioral domains.

## Supporting Information

Table S1 Tract-specific diffusivity values for autism spectrum disorder and control children. ASD = autism spectrum disorders; HC = healthy control; Rt = right; Lt = left; CCMj = forceps major, corpus callosum; CCMn = forceps minor, corpus callosum; CR = superior corona radiata; UF = uncinate fasciculus; IFOF = inferior-fronto-occipital fasciculus; SLF = superior longitudinal fasciculus; ILF = inferior longitudinal fasciculus; diffusivity units = mm2/s×10−3. *significant following multiple comparison correction.
(TIFF)

Table S2 Tract-specific diffusivity values for autism spectrum disorder and control adolescents. ASD = autism spectrum disorders; HC = healthy control; Rt = right; Lt = left; CCMj = forceps major, corpus callosum; CCMn = forceps minor, corpus callosum; CR = superior corona radiata; UF = uncinate fasciculus; IFOF = inferior-fronto-occipital fasciculus; SLF = superior longitudinal fasciculus; ILF = inferior longitudinal fasciculus; diffusivity units = mm2/s×10−3. *significant following multiple comparison correction.
(TIFF)
