# Supplementary figures and images for: Impaired Structural Connectivity of Socio-Emotional Circuits in Autism Spectrum Disorders: A Diffusion Tensor Imaging Study
Source: PLoS One. 2011 Nov 23;6(11):e28044. doi: 10.1371/journal.pone.0028044 (PMC3223195; doi:10.1371/journal.pone.0028044)

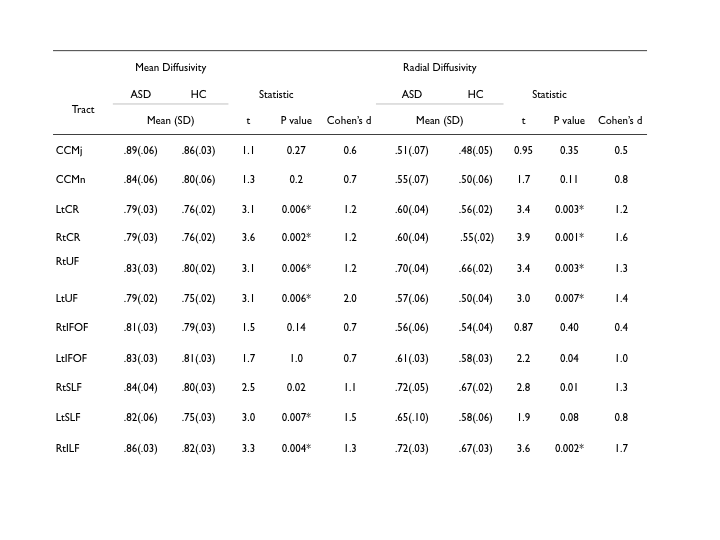

Supplement: Table S1 — Tract-specific diffusivity values for autism spectrum disorder and control children. ASD = autism spectrum disorders; HC = healthy control; Rt = right; Lt = left; CCMj = forceps major, corpus callosum; CCMn = forceps minor, corpus callosum; CR = superior corona radiata; UF = uncinate fasciculus; IFOF = inferior-fronto-occipital fasciculus; SLF = superior longitudinal fasciculus; ILF = inferior longitudinal fasciculus; diffusivity units = mm2/s×10−3. *significant following multiple comparison correction. (TIFF) [file pone.0028044.s001.tiff]

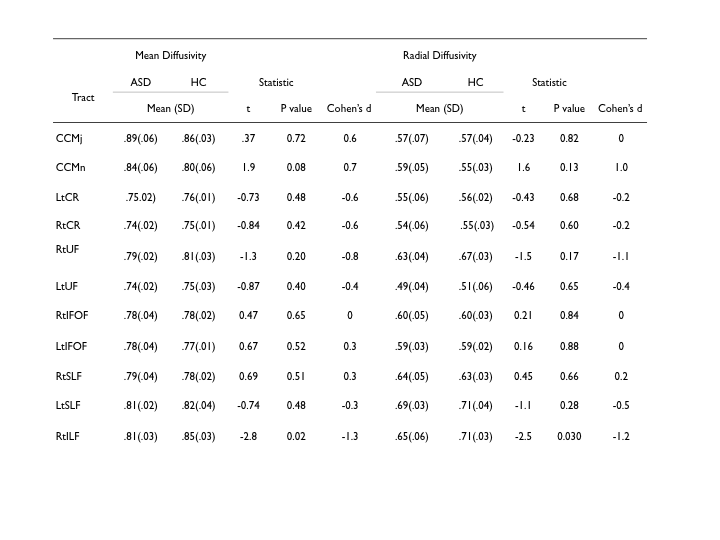

Supplement: Table S2 — Tract-specific diffusivity values for autism spectrum disorder and control adolescents. ASD = autism spectrum disorders; HC = healthy control; Rt = right; Lt = left; CCMj = forceps major, corpus callosum; CCMn = forceps minor, corpus callosum; CR = superior corona radiata; UF = uncinate fasciculus; IFOF = inferior-fronto-occipital fasciculus; SLF = superior longitudinal fasciculus; ILF = inferior longitudinal fasciculus; diffusivity units = mm2/s×10−3. *significant following multiple comparison correction. (TIFF) [file pone.0028044.s002.tiff]
